# Confirmation of *Xylella fastidiosa* (Lysobacterales: Lysobacteraceae) transmission by 8 leafhopper species present in coffee plantations in Costa Rica

**DOI:** 10.1093/jisesa/ieag068

**Published:** 2026-07-03

**Authors:** Laura Garita, Ana Mariel Zúñiga-Pereira, Andrés Arias-Paco, William Villalobos, Sofia Carvajal-Rojas, Jose Pablo Álvarez-Mora, Luis Enrique Chaves-González, Isaías Abdallah, Carolina Godoy, Phyllis G Weintraub, Mauricio Montero-Astúa, Carlos Chacón-Díaz

**Affiliations:** Centro de Investigación en Biología Celular y Molecular, Universidad de Costa Rica, San Pedro, San José, Costa Rica; Centro de Investigación en Biología Celular y Molecular, Universidad de Costa Rica, San Pedro, San José, Costa Rica; Centro de Investigación en Enfermedades Tropicales, Facultad de Microbiología, Universidad de Costa Rica, San Pedro, San José, Costa Rica; Escuela de Agronomía, Universidad de Costa Rica, San Pedro, San José, Costa Rica; Museo de Insectos, Centro de Investigación en Protección de Cultivos, Universidad de Costa Rica, San Pedro, San José, Costa Rica; Centro de Investigación en Biología Celular y Molecular, Universidad de Costa Rica, San Pedro, San José, Costa Rica; Centro de Investigación en Biología Celular y Molecular, Universidad de Costa Rica, San Pedro, San José, Costa Rica; Centro de Investigación en Enfermedades Tropicales, Facultad de Microbiología, Universidad de Costa Rica, San Pedro, San José, Costa Rica; Centro de Investigación en Enfermedades Tropicales, Facultad de Microbiología, Universidad de Costa Rica, San Pedro, San José, Costa Rica; Centro de Investigación en Enfermedades Tropicales, Facultad de Microbiología, Universidad de Costa Rica, San Pedro, San José, Costa Rica; Escuela de Ciencias Exactas y Naturales, Universidad Estatal a Distancia, Sabanilla, San José, Costa Rica; Agricultural Research Organization, Gilat Research Center, D.N. Negev, Israel; Centro de Investigación en Biología Celular y Molecular, Universidad de Costa Rica, San Pedro, San José, Costa Rica; Escuela de Agronomía, Universidad de Costa Rica, San Pedro, San José, Costa Rica; Centro de Investigación en Enfermedades Tropicales, Facultad de Microbiología, Universidad de Costa Rica, San Pedro, San José, Costa Rica

**Keywords:** Leafhopper, sharpshooter, transmission, vector, *Xylella fastidiosa*

## Abstract

*Xylella fastidiosa* Wells et al. 1987 is a vector-transmitted plant pathogen of economic importance that is widespread throughout coffee plantations in Costa Rica. Previous studies have shown the existence of a broad diversity of leafhoppers present in different coffee producing regions in Costa Rica. The presence of leafhoppers in coffee plantations with the presence of *X. fastidiosa* poses their role as potential vectors. To our knowledge, the vector status of leafhoppers present in coffee plantations in Costa Rica has remained unsettled. This work confirmed 8 species of leafhoppers as vectors of *X. fastidiosa* related to coffee in Costa Rica. Insects were collected in field in the northern part of the Costa Rican Central Valley, and leafhoppers were identified, classified, and grouped for further analysis. All the confirmed vector species belong to the subfamily Cicadellinae (Hemiptera: Auchenorrhyncha), known as sharpshooters. The ability of these specimens to survive on coffee plants for a 72-h period was assessed. *In planta* transmission assays were performed on *X. fastidiosa*-free coffee plants under greenhouse conditions. *X. fastidiosa* was detected in plants after one and a half years, confirming the vector status of leafhoppers. Positive plants remained asymptomatic throughout the experiment. The implication of these species as vectors strengthens surveillance actions in local and international contexts.

## Introduction

*Xylella fastidiosa* is a vector-transmitted bacterial plant pathogen of economic importance (Sicard et al. 2018), found in the xylem of a broad range of host plants. In Costa Rica, 2 subspecies of *X. fastidiosa* co-exist: endemic *X. fastidiosa* subsp. *fastidiosa* (*Xff*), and *X. fastidiosa* subsp. *pauca* (*Xfp*), introduced from South America (Vanhove et al. 2019, Castillo et al. 2020). *Xff* isolates show broad genetic diversity (Castillo et al. 2020), whereas in *Xfp*, all characterized isolates are within the ST53 clade (Nunney et al. 2014, Giampetruzzi et al. 2017).*X. fastidiosa* is present throughout coffee producing regions within the Central Valley (Rodríguez et al. 2001, Garita-Cambronero et al. 2008, Montero-Astúa et al. 2008a).

Despite *X. fastidiosa*’s great pathogenic potential, in Costa Rica, *X. fastidiosa* colonized coffee plants are, in most cases, asymptomatic or present a variety of mild symptoms, such as leaf curling, leaf malformation, internode shortening, and premature loss of leaves described as “crespera disease” (Montero-Astúa et al. 2008a). As reported for *X. fastidiosa* diseases, symptoms develop from a local focal point in the plant but can spread systematically (Hopkins and Purcell 2002). Severe symptoms, such as leaf scorch, have not been observed in symptomatic coffee plants in Costa Rica (Rodríguez et al. 2001, Montero-Astúa et al. 2008a). There is scarce information regarding the potential negative impact of “crespera” on coffee production with colonized versus healthy coffee plants (Solórzano et al. 2001). *X. fastidiosa* is also present in citrus and avocado trees used as shade plants in coffee plantations (Aguilar et al. 2005, Montero-Astúa et al. 2008b).

Research in Costa Rica has focused mainly on *X. fastidiosa* genetic characterization (Nunney et al. 2014, Giampetruzzi et al. 2017, Castillo et al. 2020) and host description as reviewed in De la Fuente et al. (2017). Vector biology dynamics in the *X. fastidiosa*-coffee pathosystem in Costa Rica is a promising and challenging field due to the diversity and potential role of specific vectors for which their biology is largely unknown. Past studies have shown that there is great diversity of hemipteran species in the families Cicadellidae (Auchenorrhyncha: Membracoidea) and Clastopteridae (Auchenorrhyncha: Cercopoidea) in coffee plantations in Costa Rica (Rojas et al. 2001, Garita-Cambronero et al. 2008); these insects represent potential *X. fastidiosa* vectors. However, the status remains unsettled and has only been preliminarily addressed (Garita et al. 2021), as well as the characterization of basic biological and epidemiological traits.

In this work, we focused on (i) evaluating the absolute and relative presence of cicadellids in a coffee plantation in the northern region of the Central Valley, (ii) determining *Xylella*-transmitting capacity of a series of leafhoppers associated with coffee in Costa Rica, and (iii) classifying and identifying vectors. These findings will contribute to a comprehensive understanding of the pathosystem and to the development of surveillance actions in local and global contexts.

## Materials and Methods

### Coffee Plant and Greenhouse Maintenance

Three-month-old Catuai variety coffee seedlings were used for *in planta* assays. Plants were kept in greenhouse facilities at the University of Costa Rica, previous to and throughout the duration of the trial (one and a half years). These plants were tested for the presence of *X. fastidiosa* and were determined to be *X. fastidiosa*-free, using Real Time Polymerase Chain Reaction (RT-PCR) method (Francis et al. 2006). Coffee plants were transplanted into individual pots (16 cm diameter and 16 cm height) with sterilized soil containing a slow-release fertilizer and treated with carboxin as a precautionary measure for root pathogens. Temperature and humidity conditions fluctuated according to outdoor conditions. Coffee plants kept in the same condition but not exposed to sharpshooters were used as a negative control for plant status for *X. fastidiosa* throughout the transmission trial.

### Field Collection of Insects

A coffee plantation in the locality of Carrizal, Alajuela province (10°04′28.71″N, 84°10′11.48″W) that had been previously tested and reported for the presence of *X. fastidiosa* (Garita-Cambronero et al. 2008), was used as the source for infected xylem-feeding leafhoppers. Twenty-five coffee plants located specifically in the collecting sites within the plantation were randomly selected and screened to confirm the presence of *X. fastidiosa*; the plants were also evaluated for crespera disease symptoms. Leafhopper collection was performed to determine absolute and relative abundance of leafhoppers, to identify and classify leafhoppers, and to serve as a source for in planta transmission setups under greenhouse conditions.

**Fig. 1.**
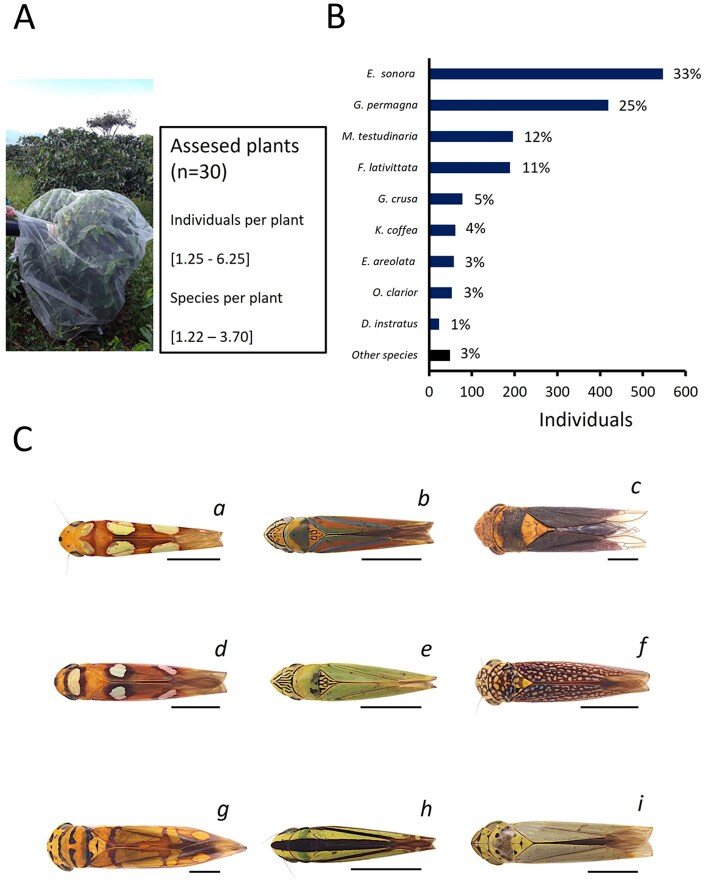
Cicadellid species and population abundances in the assessed coffee plantation. A) Thirty plants were selected to assess the absolute abundance of individuals and species per plant. B) Relative abundance of field-collected species of cicadellids. C) Photographic representation of the dorsal habitus of leafhopper vectors of *X*. *fastidiosa* in coffee plantations in Costa Rica. a) *Erythrogonia sonora*, b) *Graphocephala crusa*, c) *Oncometopia clarior*, *d*) *Erythrogonia areolata*, e) *Graphocephala permagna*, *f*) *Macugonalia testudinaria*, *g*) *Dilobopterus instratus*, *h*) *Fusigonalia lativittata*, *i*) *Kapateira coffea*. Scale bar = 2 mm.

Field sampling was performed between July 2020; and January 2021; (see Table 1 for specific collection dates). Leafhoppers were collected by vacuum sampling coffee plants (50 m in a row). The same coordinates were used for different sampling dates, but not necessarily the same rows. An inverted-flow commercial hand leaf blower (Stihl SH 86C) with custom made nylon-mesh bags (16 cm diameter × 50 cm long) was used for insect sampling. Collection bags were kept in a cooler with high humidity while being transported to the laboratory. Two collected bags per visit were used for leafhopper identification and relative abundance determination. The rest of the collected material was used immediately after grouping per species for in planta transmission assays in greenhouse conditions. To determine the absolute abundance of cicadellids per coffee plant at the site of study, insects were vacuumed after placing a mesh bag over the whole individual plant; they were neither able to fly away during vacuuming nor were they collected from an adjacent plant (Fig. 1A). This collection method was applied to thirty plants selected randomly within the collection site. Permits for biological samples and research on genetic resources were granted by the “Comisión Institucional de Biodiversidad” (Institutional Biodiversity Committee, University of Costa Rica; Resolution Numbers 225, 344, 367).

**Table 1.** *X. fastidiosa* detection (number of *X. fastidiosa-*positive pools/total number of pools tested) in field-collected pooled cicadellid species throughout sampling dates

	Collection date^a^														
	26-Jun	3-Jul	4-Aug	11-Aug	25-Aug	1-Sep	8-Sep	15-Sep	22-Sep	29-Sep	13-Oct	20-Oct	27-Oct	10-Nov	26-Jan
***Graphocephala permagna***	–	2/2	3/3	2/2	4/4	2/2	2/2	2/2	4/4	2/2	1/1	2/2	1/1	1/1	–
***Graphocephala crusa***	–	1/1	–	0/1	0/2	0/1	–	0/1	0/1	–	1/1	1/1	1/1	1/1	–
***Graphocephala bivittata***	–	–	–	–	–	1/1	1/1	2/2	1/1	–	–	-	–	–	–
***Grpahocephala riverae***	–	–	–	–	–	–	–	–	–	1/1	–	–	-	–	–
***Erythrogonia sonora***	–	0/2	3/7	5/5	2/3	2/3	0/1	2/2	0/1	1/1	1/2	1/1	1/2	1/1	1/1
***Erythrogonia areolata***	1/1	0/1	0/1	0/1	0/1	0/1	0/1	1/1	1/1	1/1	0/1	0/1	0/1	0/1	0/1
***Macugonalia testudinaria***	1/2	0/2	1/1	1/2	2/2	1/1	1/1	0/1	0/1	1/1	1/2	1/2	1/3	0/1	–
***Fusigonalia lativittata***	1/1	2/3	–	1/1	1/1	1/1	–	–	–	–	–	1/1	0/1	2/2	–
***Kapateira coffea***	–	2/2	–	–	1/1	1/1	–	1/1	1/1	0/1	0/1	1/1	0/1	1/1	–
***Oncometopia clarior***	0/2	–	0/1	1/1	1/1	1/2	0/1	–	0/1	0/1	0/1	–	–	–	–
***Dilobopterus instratus***	0/1	1/1	–	–	–	1/1	–	1/1	1/2	1/1	1/1	–	0/1	–	1/1

(–) Analysis not performed due to the absence of species on the collection date.

a Collection dates are represented between June 2020 and January 2021.

### Identification and Classification of Leafhoppers

The specimens were separated into morphospecies, and the number of individuals per species was recorded and used for *in planta* assays. For taxonomic identification, a series of 4 to 5 males of each species was selected, and their genitals were dissected, placing the abdomen in 10% potassium hydroxide for 24 h at room temperature. Then they were washed in water for 3 min and subsequently deposited in microvials with glycerin (Oman, 1949). Representative specimens were mounted and identified by Carolina Godoy and Andrés Arias-Paco, according to previous descriptions and taxonomic keys (Melichar 1926; Medler 1963; Young 1977; Nielson and Godoy 1995; Godoy et al. 2006; Godoy and Villalobos 2006; Rakitov 2016). The voucher specimens were deposited in the collection of the Insect Museum at the University of Costa Rica (MIUCR), codes MUCR 0002392 to MUCR 0002428.

### Detection of *X. fastidiosa* in Leafhoppers

DNA extraction was carried out following an accessory protocol intended for the purification of total DNA from insects using the DNeasy Blood and Tissue DNA extraction kit (Qiagen, Germany). Briefly, the head of individual or pooled insects were macerated in liquid nitrogen, lysis was performed for 3 h at 52 °C, and column purification and isolation were performed according to the manufacturer’s protocol. Specific detection of *X. fastidiosa* was performed by PCR, expecting a 733-bp amplicon representing the *rpoD* gene. PCR reaction and conditions using primer pairs RST31 (5′-GCGTTAATTTTCGAAGTGATTCGATTGC-3′) and RST33 (5′-CACCATTTCGTATCCCGGTG-3′) were performed as described previously (Minsavage et al. 2025). Samples were tested using direct DNA extraction as a template and a 1/10 dilution. DNA from an endemic *Xff*, XF-68 isolate (Castillo et al. 2020), was used as a positive control.

### Detection of *X. fastidiosa* from Plant Material

DNA extraction was performed with DNeasy Plant Mini Kit-based extraction (Qiagen), following the manufacturer’s instructions. Detection of *X. fastidiosa* from plant material was carried out following established protocols. Conventional PCR for the detection of *X. fastidiosa* was performed as described previously. Real-time PCR tests for the specific detection of *X. fastidiosa* were performed by PCR amplification of a 201-bp amplicon representing a conserved hypothetical protein (XF_1717). PCR reaction and conditions using primer pairs HL5 (5′-AAGGCAATAAACGCGCACTA-3′) and HL6 (5′-GGTTTTGCTGACTGG CAACA-3′) were performed as described previously using the SYBR Green version (Francis et al. 2006). Samples were considered positive according to established parameters such as the generation of an exponential amplification curve and a specific melting peak within an 83 to 85 °C range. DNA from *Xff*, XF-68 isolate, was used as a positive control.

### Plant Survival Test and Transmission Assays

Sharpshooters were aspirated by species in groups of 10 individuals; the number of female and male individuals per group was not recorded. The number of setups per species depended on insect availability and the number for each sampling date. Each group was introduced to a coffee plant, 20 cm tall, covered with a nylon mesh bag; the plant was left with the insects for an inoculation access period (IAP) of 72 h. At the end of the IAP, the mesh bag was removed, and the number of live and dead leafhoppers was counted. All insects were then placed in 95% EtOH and frozen at −80 °C for further testing. A total of 162 coffee plant trials were conducted across the sampling dates. Pooled insects were tested for *X. fastidiosa*, as mentioned before. Coffee plants exposed to insects that tested negative for *X. fastidiosa* were discarded. Plants inoculated with *X. fastidiosa*-positive pooled insects were kept in greenhouse conditions for the remainder of the experimental period. These plants were monitored for symptoms and tested for *X. fastidiosa* one and a half years after inoculation. At this point, the plants measured 50 cm in height. To assess the potential spread of *X. fastidiosa* within the plant, positive plants were tested at 2 different points, 10 and 20 cm above the initial inoculation site, following previously described PCR methods.

## Results

### Cicadellids Present in Collecting Sites Are Diverse and Abundant

We confirmed that *X. fastidiosa* is highly prevalent in coffee plants at the chosen study site. Sixty percent of randomly sampled plants tested positive for *X. fastidiosa* (15 out of 25), but fewer than 1% showed crespera symptoms, confirming a high presence of the bacteria in the sampling area. To ensure our sampling represented insect populations interacting with coffee, collection sites were located away from existing field boundaries that host numerous plant species and insect specimens. Despite monoculture practices, cicadellids showed an absolute abundance of 1.25 to 6.25 individuals and 1.22 to 3.70 species per plant (Fig. 1A). Nine Cicadellidae species accounted for 97% of the relative abundance and diversity, all belonging to the subfamily Cicadellinae (Hemiptera: Auchenorrhyncha), known as sharpshooters *Dilobopterus instratus* (Fowler, 1899), *Erythrogonia areolata* (Signoret, 1853), *Erythrogonia sonora*  Melichar, 1926, *Fusigonalia lativittata* (Fowler, 1900), *Graphocephala crusa* Godoy, 2006, *Graphocephala permagna*  Nielson & Godoy, 1995, *Kapateira coffea* Godoy, 2006, *Macugonalia testudinaria* (Fowler, 1899), and *Oncometopia clarior* (Walker, 1851) (Fig. 1B and C and Supplementary Fig. S1). Additionally, 11 species made up the remaining 3%, some of which are still unidentified or classified.

**Fig. 2.**
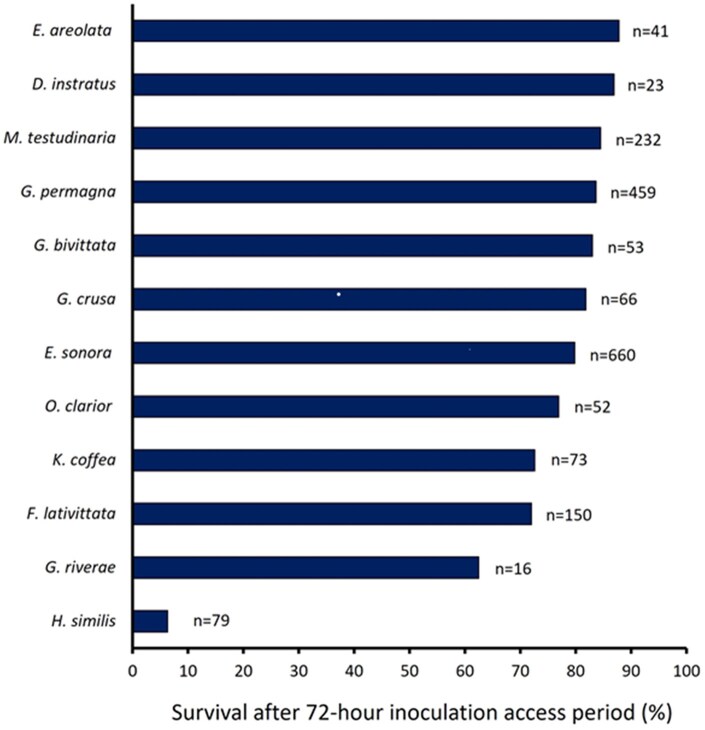
Survival rate of the tested cicadellid species was evaluated 72 h after inoculation during the inoculation access period on the experimental coffee plants.

Differences in the field abundance of species and detection of *X. fastidiosa* were observed across sampling dates (Table 1). Species from the same genus showed variations in abundance and *X. fastidiosa* positivity over time. *Graphocephala permagna* was more abundant and present at all sampling dates; all pooled samples tested positive for *X. fastidiosa*. In contrast, *G. crusa* was less abundant and showed intermittent presence of *X. fastidiosa*. Other *Graphocephala* species, *G. bivittata* and *G. riverae* (Godoy and Villalobos, 2006), were only present to a lesser extent and on specific collection dates (Table 1). The same pattern was observed for *E. sonora*, which was more abundant and harbored more *X. fastidiosa* than *E. areolata*. *M. testudinaria*, *F. lativittata*, *K. coffea*, *O. clarior*, and *D. instratus* appeared intermittently throughout the collection dates (Table 1). We assessed the survival of the collected cicadellids on coffee plants over 72 h IAP. Except for *Hortensia similis* (Walker, 1851) (Cicadellidae: Cicadellinae), mainly found in herbaceous vegetation within coffee plantations, the other species exhibited survival rates above 70% on coffee plants (Fig. 2), suggesting a potential for successful *X. fastidiosa* transmission. Additional field observations and greenhouse experiments showed that cicadellids not only survived on coffee but also fed on it, as indicated by honeydew secretion.

### Confirmation of *X. fastidiosa* Transmission to Coffee Plants by Cicadellid Species

**Fig. 3.**
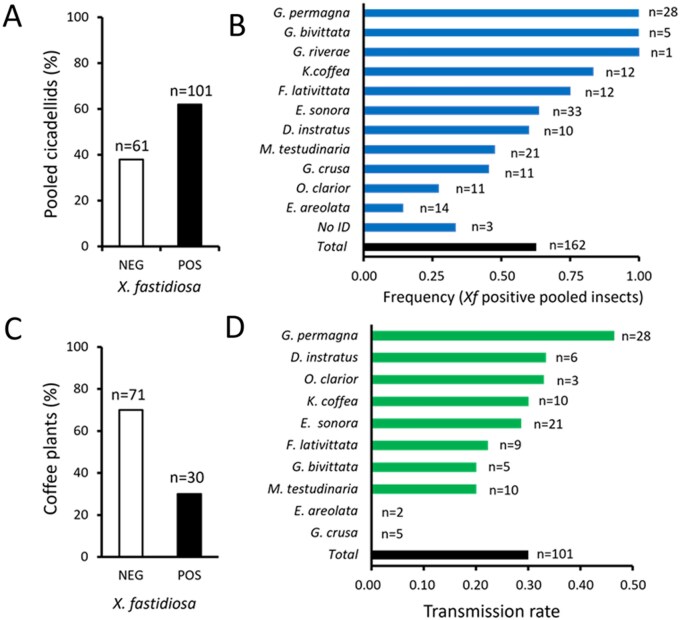
Confirmation of 8 *X. fastidiosa* vectors related to coffee in Costa Rica. Field-collected sharpshooters were identified and grouped by species. A) Sixty percent of the pooled sharpshooters used in the transmission assays were positive for *X. fastidiosa*; negative setups were discarded for further analysis. B) *X. fastidiosa* positivity frequency for pooled sharpshooters used for transmission assays. C) Thirty percent of the tested coffee plants became *X. fastidiosa* positive after one and a half years. D) *X. fastidiosa* vector transmission frequency of different species of sharpshooters related to coffee in Costa Rica.

A total of 162 transmission assays were performed on coffee plants in greenhouse conditions. After a 72-h IAP, individuals for each transmission setup were recovered and tested as a pooled sample. *X. fastidiosa* was detected in 60% of pooled samples (Fig. 3A), confirming the abundant presence of *X. fastidiosa* throughout the sampling dates. *G. permagna* and *E. sonora* accounted for 50% of the total *X. fastidiosa* positive pooled cicadellid samples. Plant setups with *X. fastidiosa* negative pooled insects (40%) were discarded for further analysis (Fig. 3A). All *Graphocephala* species, *G. permagna*, *G. bivittata*, and *G. riverae* pooled samples used for in planta transmission assays were positive for *X. fastidiosa* (Fig. 3B), suggesting that at least 1 individual per pool was *X. fastidiosa* positive. The other species showed different frequency for *X. fastidiosa* detection in pooled samples.

Coffee plants were tested for *X. fastidiosa* one and a half years after cicadellid exposure. Coffee plants did not develop symptoms related to “crespera” disease throughout the duration of the assay. Thirty percent of the coffee plants became *X. fastidiosa* positive (Fig. 3C), confirming the vector status of cicadellid species present in coffee plantations in Costa Rica that can successfully transmit *X. fastidiosa* to coffee. Forty-three percent of *X. fastidiosa* positive plants were exposed to *G. permagna*. In contrast, no positive plants were detected concerning *G. crusa* and *E. areolata*. *G. permagna* showed the highest transmission rate (0.46), followed by *D. instratus* (0.33) and *K. coffea* (0.30) (Fig. 3D).

**Fig. 4.**
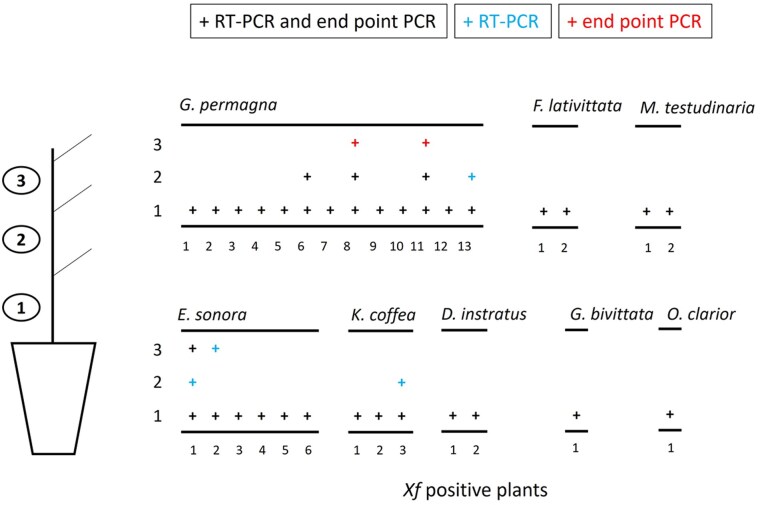
Evaluation of vector-transmitted *X. fastidiosa* dispersal in coffee plants. Dispersal of *X. fastidiosa* throughout coffee plants was determined by evaluating the presence of *X. fastidiosa* at 10 and 20 cm (marked as 2 and 3, respectively) from the original feeding location (marked as 1). Coffee plants did not show symptoms throughout the duration of the assay.

Positive coffee plants were evaluated for *X. fastidiosa* dispersal from the vector inoculation point. In 4/30 positive plants, *X. fastidiosa* was detected at 10 and 20 cm from the inoculation point; in 3/30 plants, it was only detected at 10 cm from the inoculation point (Fig. 4). In the remaining 23 plants, *X. fastidiosa* was only detected at the insect feeding area 18 months after exposure to insects. *G. permagna*, *E. sonora*, and *K. coffea* were the vector species involved in transmission with *X. fastidiosa* dispersal within the plant.

## Discussion

In this study, we confirmed *X. fastidiosa* transmission to coffee plants by 8 vector species (Fig. 3), all belonging to the Cicadellinae subfamily, known as sharpshooters: *D. instratus*, *E. sonora*, *F. lativittata*, *G. bivittata*, *G. permagna*, *K. coffea*, *M. testudinaria*, and *O. clarior*. We report transmission rates (for pools of 10 individuals) from field-collected insects to coffee that range from 0% to 46% (Fig. 3C), which agrees with transmission efficiencies reported in several trials performed by different research groups for different vector species (Redak et al. 2004, Cornara et al. 2019). All genera except for *Kapateira* have been reported as main *X. fastidiosa* vectors in North and or South America (Cornara et al. 2019). Most of these new vector species are circumscribed to the Central America region (Medler 1963, Young 1977, Nielson and Godoy 1995, Godoy et al. 2006) and are different from vector species present in coffee in South America (Silva et al. 2007, Marucci et al. 2008). *K. coffea* was described as a species present in Costa Rica associated with coffee and citrus, with the potential to harbor *X. fastidiosa* (Godoy et al. 2006). To our knowledge, this is the first report of a species of the *Kapateira* genus as a *X. fastidiosa* vector. We were able to confirm vector status of *G. permagna* and *E. sonora*, 2 species that are abundant in coffee plantations and have tested positive for *X. fastidiosa* since the first report of the disease in coffee in Costa Rica in the 1990s (Rodríguez et al. 2001).

*G. permagna* was the most abundant leafhopper, present throughout sampling dates, can feed exclusively on coffee, has the highest percentage of positive *X. fastidiosa* field-collected specimens, and showed the highest transmission rates to coffee plants (46%), evidencing an important role in the pathosystem. In this case, the most abundant leafhopper correlates with the best transmission rates; however, this is not necessarily the case (Almeida et al. 2005). The other confirmed species, as *X. fastidiosa* vectors to coffee in this study, *D. instratus*, *K. coffea*, *E. sonora*, *F. lativittata*, *G. bivittata, M. testudinaria*, and *O. clarior*, showed variation in feeding on coffee, abundance, transmission rates, and positivity for *X. fastidiosa*, contributing also to pathogen dispersal within coffee plantations.

Leafhoppers from this study were commonly found on coffee plants and can survive and feed for at least 72 h on coffee plants in greenhouse conditions. This biological trait should increase the probability of infection (Almeida et al. 2005); however, it does not necessarily correlate with the efficiency of transmission as seen for *H. vitripennis* in Pierce Disease (Almeida and Purcell 2003). Further studies are required to understand specific biological traits associated with these vector populations that sustain *X. fastidiosa* dispersion in coffee plantations. As reported previously, there is no vector specificity to *X. fastidiosa* strains; practically all Cicadellinae are potential vectors (Almeida and Nunney 2015). More efforts are required to demonstrate that 1 individual can hold and transmit more than 1 *X. fastidiosa* species and the impact of this vector–bacteria condition on the pathosystem.

Only for *K. coffea*, there is information on its life cycle, which was completed exclusively on coffee in growth chamber experiments (Godoy et al. 2006). The description of each vector life cycle, flight performance, and the factors that affect vector behavior, as reported for *Philaenus spumarius* (Lago et al. 2021), has not been systematically studied. Relevant information regarding feeding behavior in relation to transmission of *X. fastidiosa* (Backus and Shih 2020), the impact of plant species as *X. fastidiosa* cryptic hosts or reservoirs, and the description of drivers of vector sustainability and diversity (Almeida et al. 2005), is missing for these vector species.

Surveillance of insect diversity in coffee plantations as an indicator of crop health may anticipate the introduction, fixation, or emplacement of specific leafhoppers that enhance disease, as seen in vineyards in California (Almeida and Purcell 2003); as well as the response of *X. fastidiosa* strains to cues that favor a shift from commensal to parasitic expression of pathogenic traits (Roper et al. 2019). Such features have been reported as drivers of *X. fastidiosa* epidemics in other latitudes.

Overall, these findings contribute to the existing knowledge gap concerning the role of leafhopper species, specifically sharpshooters, in the transmission of *X. fastidiosa*–coffee pathosystem in Costa Rica. It also gives information on *X. fastidiosa–*vector dynamics to monitor potential abiotic and biotic drivers, such as climate change, that can cause reduction, fixation, or introduction of leafhoppers into the pathosystem, addressing issues of major challenges to overcome in understanding *X. fastidiosa* diseases (De la Fuente et al. 2024). The inclusion of these specific sharpshooters as vectors strengthens surveillance actions, in a local context, and international plant trade procedures for mitigation purposes.

## Supplementary Material

ieag068_Supplementary_Data
