# Supplementary figures and images for: Confirmation of Xylella fastidiosa (Lysobacterales: Lysobacteraceae) transmission by 8 leafhopper species present in coffee plantations in Costa Rica
Source: J Insect Sci. 2026 Jul 3;26(4):ieag068. doi: 10.1093/jisesa/ieag068 (PMC13331132; doi:10.1093/jisesa/ieag068)

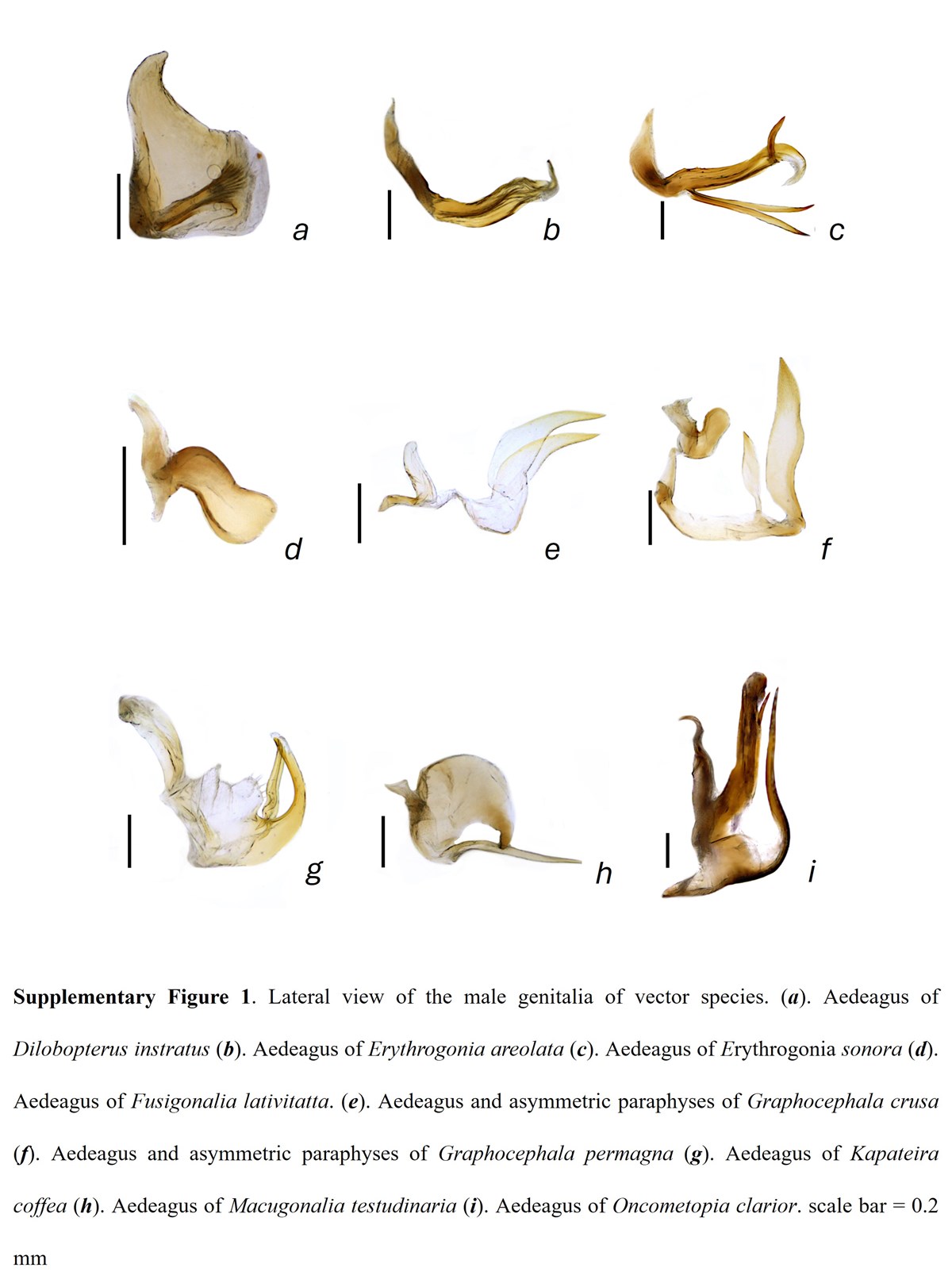

Supplement: ieag068_Supplementary_Data [file ieag068_supplementary_data.jpeg]
